# Pregnancy after fertility preservation and multimodal therapy including intensity-modulated radiotherapy for recurrent vulvar cancer: A case report

**DOI:** 10.1007/s13691-026-00855-5

**Published:** 2026-03-11

**Authors:** Kosuke Murakami, Kiko Yamamoto, Takuya Uehara, Yukinori Matsuo, Noriomi Matsumura

**Affiliations:** 1 Department of Obstetrics and Gynecology, Kindai University Faculty of Medicine, 1-14-1 Miharadai, Minami-ku Sakai, Osaka 590-0197 Japan; 2 Department of Radiation Oncology, Kindai University Faculty of Medicine, Sakai, Osaka Japan

**Keywords:** Vulvar cancer, Fertility preservation, Intensity-modulated radiotherapy, Hormone replacement therapy, Embryo transfer

## Abstract

Advanced or recurrent vulvar cancer in women of reproductive age is extremely rare, and the effects of radiotherapy (RT) on uterine and ovarian function in such cases remain poorly understood. Here, we report the case of a 36-year-old woman with recurrent vulvar cancer after initial surgery. She strongly desired fertility preservation, so before undergoing resection of the recurrent lesion and inguinal lymphadenectomy, she underwent ovarian stimulation using a random-start progestin-primed ovarian stimulation (PPOS) protocol, and embryos were cryopreserved. Because of the high risk of recurrence, the patient underwent adjuvant intensity-modulated radiotherapy (IMRT) postoperatively. Three months after irradiation, ovarian function was lost; however, endometrial regrowth and withdrawal bleeding were observed with oral administration of norgestrel and ethinylestradiol. Despite significant vaginal and cervical atrophy, frozen-thawed embryo transfer was successfully performed in a hormone replacement cycle. Pregnancy was achieved with the second blastocyst transfer. Unfortunately, the patient subsequently developed acute myeloid leukemia, and continuation of the pregnancy was no longer feasible. This case suggests that IMRT may minimize scatter radiation to the uterine cavity, allowing for partial preservation of uterine function. Even in cases of advanced or recurrent vulvar cancer, fertility preservation strategies should be considered when appropriate.

## Introduction

Vulvar cancer is a rare malignancy, accounting for approximately 5% of all gynecologic cancers [1]. However, the incidence of advanced or recurrent cases in women of reproductive age is on the rise [2]. For advanced vulvar cancer, standard treatment includes radical vulvectomy, and in cases with a high risk of recurrence, adjuvant radiotherapy (RT) may be added [1]. However, the impact of such treatment on uterine and ovarian function in reproductive-age women has not been fully elucidated.

With an increasing number of young women expected to survive long-term after cancer treatment, the effects of therapy on fertility have become a critical issue. Pelvic RT poses risks such as depletion of the primordial follicle pool and premature ovarian insufficiency [3]. In addition, impaired uterine blood flow can lead to endometrial atrophy and decreased uterine volume and elasticity, resulting in reduced fertility [3, 4]. Therefore, efforts are being made to minimize radiation exposure to reproductive organs in young female patients receiving RT. Technological advancements, including intensity-modulated RT (IMRT) and image-guided RT (IGRT), have enabled more precise control of the irradiation field and reduced unnecessary radiation to surrounding normal tissues [5, 6]. With these techniques, in cases of diseases that do not directly involve the uterus, such as vulvar, anal, or rectal cancer, it is theoretically possible to minimize scatter radiation to the uterus. However, there is a lack of clinical evidence on how much these methods contribute to preserving uterine function, and detailed evaluations from the perspective of fertility preservation remain scarce.

In this report, we present a rare case of recurrent vulvar cancer treated with multimodal therapy including surgery and IMRT. Although ovarian function was lost, endometrial proliferation was achieved with oral administration of norgestrel and ethinylestradiol, and pregnancy was successfully established by frozen-thawed embryo transfer.

### Case report

**Fig. 1:**
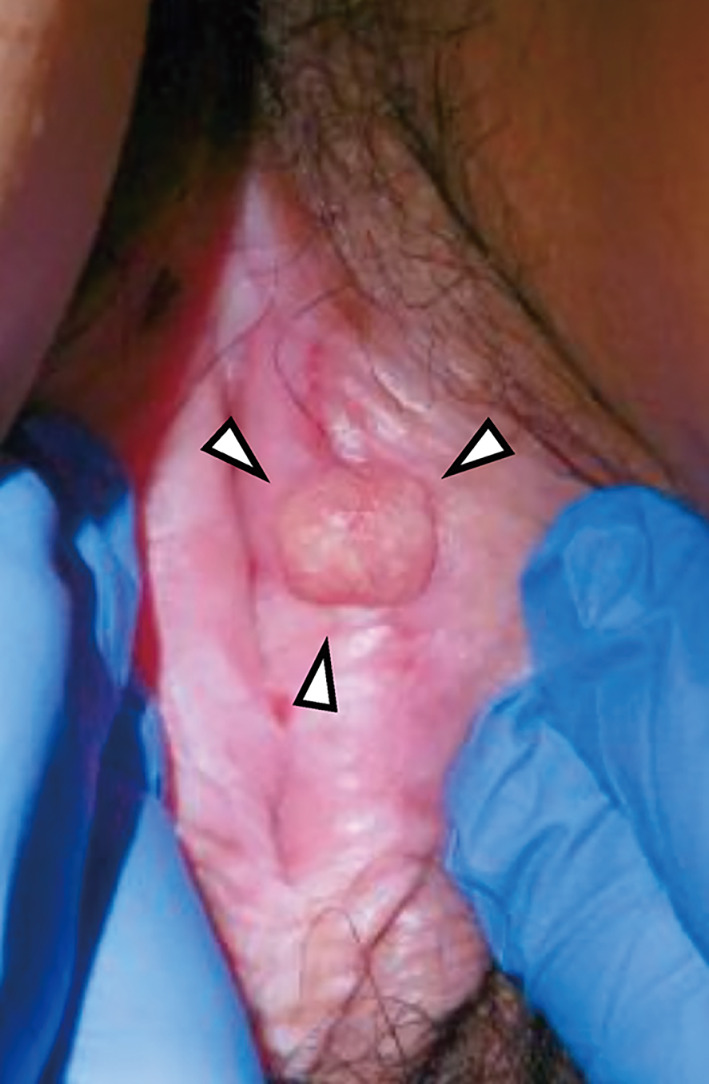
Vulvar tumor at initial presentation. A 1.5 cm mass is observed on the left labia majora (white arrowhead)

The patient was a nulligravid, nulliparous woman. She was 147 cm tall and weighed 55 kg, with a body mass index (BMI) of 25.5 kg/m^2^. She had no notable medical history. At the age of 32, she noticed a 1.5 cm mass on her left labia majora. A biopsy confirmed vulvar squamous cell carcinoma (Fig. 1). She underwent wide local excision and left inguinal lymph node biopsy. Postoperative pathological diagnosis was International Federation of Gynecology and Obstetrics (FIGO) stage IA (pT1aN0M0) squamous cell carcinoma with negative margins, and no adjuvant therapy was given. She was followed up without recurrence.

**Fig. 2:**
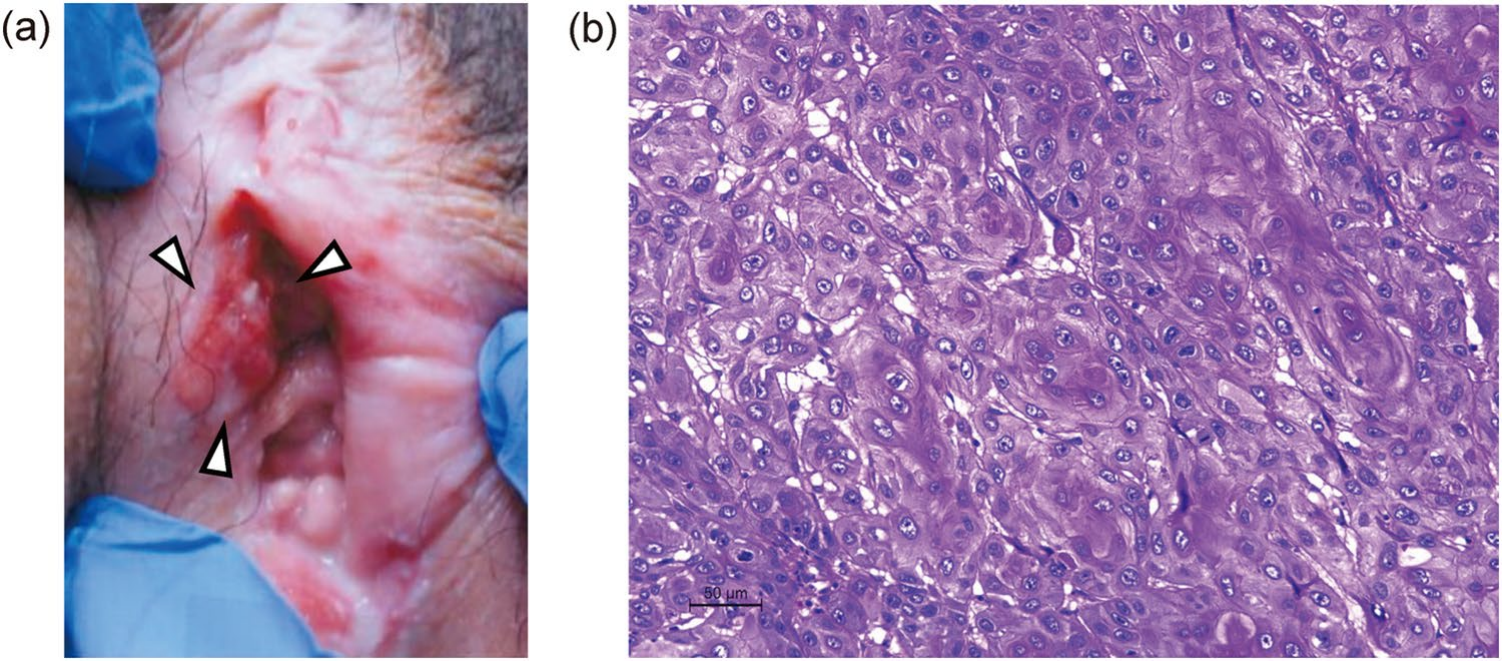
Recurrent vulvar tumor. **A**. A mass is observed on the right labia (white arrowhead). **B**. Histopathological image of the resected tumor. Hematoxylin and eosin staining; scale bar: 50 µm

At age 35, she got married and began standard infertility treatment six months later. However, at age 36, a recurrent vulvar mass was observed, which gradually enlarged (Fig. 2a). A biopsy confirmed recurrence of vulvar squamous cell carcinoma. At this time, the patient strongly desired fertility preservation, and ovarian stimulation using a progestin-primed ovarian stimulation (PPOS) protocol was initiated. Anti-Müllerian hormone (AMH) level was 5.22 ng/mL. Although a random-start stimulation protocol was initially considered, the patient’s menstrual period started just before treatment initiation, allowing conventional stimulation to begin on day 3 of menstruation. After 10 days of recombinant follicle-stimulating hormone (rFSH) 150 IU administration per day, follicular growth was observed, and ovulation was triggered using 5,000 IU of human chorionic gonadotropin (hCG). Transvaginal oocyte retrieval was performed 34 hours later, yielding 9 mature oocytes. Fertilization was achieved through conventional insemination, resulting in cryopreservation of two blastocysts and one morula.

**Fig. 3:**
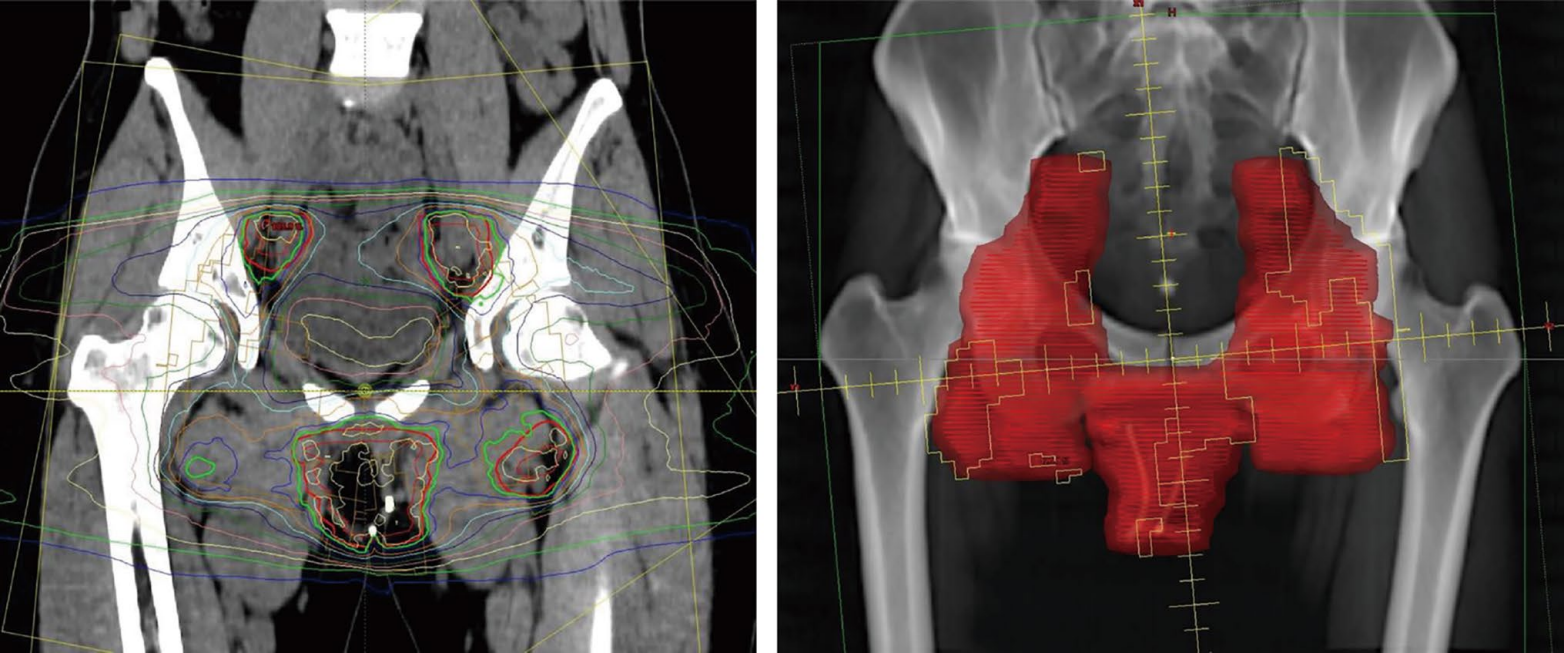
Radiation plan using intensity-modulated radiotherapy. The irradiation fields included the vulva, bilateral inguinal lymph nodes, and pelvic lymph node areas

Subsequently, the patient underwent radical vulvectomy and bilateral inguinal lymphadenectomy for the recurrent lesion. Pathological examination again confirmed recurrent vulvar squamous cell carcinoma (Fig. 2b). Metastasis was found in the right inguinal lymph nodes, indicating a high risk of further recurrence. Adjuvant IMRT was administered. The irradiation field included the vulva, bilateral inguinal lymph nodes, and pelvic lymph node regions as the clinical target volume, adding set-up margins of 5.0 mm for the planning target volume (PTV), with a total dose of 50.4 Gy (1.8 Gy × 28 fractions) (Fig. 3). Four full arcs of volumetric modulated arc therapy technique were applied with 6 MV flattening filter-free photon beams using Halcyon linac (Varian Medical Systems Inc., Palo Alto, CA). The prescribed dose was normalized as 50.4 Gy to 50% of the PTV. To minimize radiation exposure to the uterus, the bladder was filled before each session. Dose–volume histogram (DVH) parameters for the uterus were evaluated using D_max_, D_mean_, V_14Gy_, and V_20Gy_. Dmax was defined as the maximum dose, and Dmean as the mean dose. V_14Gy_ and V_20Gy_ were defined as the volumes of the uterus receiving ≥14 Gy and ≥20 Gy, respectively. The D_max_, D_mean_, V_14Gy_, and V_20Gy_ of the uterus were 25.1 Gy, 5.9 Gy, 1.8 cc, and 0.1 cc, respectively. DVH parameters for the ovaries were evaluated using D_max_, D_mean_, V_5Gy_, and V_14Gy_, where V_5Gy_ was defined as the volume receiving ≥5 Gy. The D_max_, D_mean_, V_5Gy_, and V_14Gy_ of the ovaries were 6.7 Gy, 3.9 Gy, 1.3 cc, and 0 cc, respectively. Radiation-induced vulvitis occurred and was managed with topical steroids and petroleum jelly. Severe vaginal atrophy was also observed, and topical estrogen cream was applied.

**Fig. 4:**
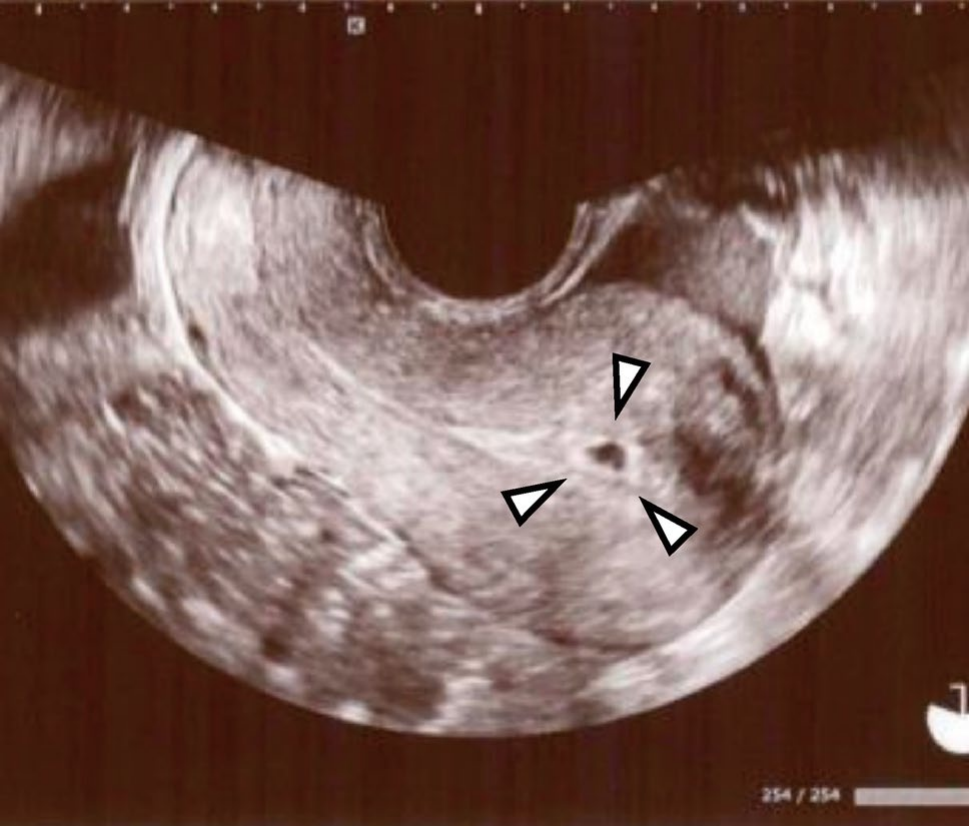
Intrauterine gestational sac

Three months after the completion of RT, hormonal assessment revealed serum estradiol below the detection limit (<10 pg/mL), FSH of 99.3 mIU/mL, and AMH below the detection limit (<0.1 ng/mL), indicating complete ovarian failure. Although uterine function was presumed impaired, oral administration of norgestrel and ethinylestradiol induced endometrial proliferation (up to 9.5 mm) and withdrawal bleeding. Despite severe vaginal and cervical atrophy, transcervical tubing was possible. Frozen-thawed embryo transfer was attempted during a hormone replacement cycle using estradiol patches (2.88 mg every other day). The first blastocyst transfer did not result in pregnancy, but the second attempt led to a confirmed intrauterine gestational sac (Fig. 4). Unfortunately, at 5 weeks of gestation, the patient was diagnosed with acute myeloid leukemia (AML). Although a causal link with RT was suspected, it remained unproven. Pregnancy could not be continued, and termination was performed at 6 weeks.

## Discussion

This is an extremely rare case in which fertility preservation therapy was performed in a patient with recurrent vulvar cancer, and following IMRT, endometrial proliferation was achieved with oral norgestrel–ethinylestradiol administration, ultimately resulting in pregnancy through frozen-thawed embryo transfer. We reviewed previous reports of pregnancies occurring after RT for non-uterine pelvic malignancies such as rectal, anal, or vulvar cancer. Case reports extracted using the PubMed search formula ((rectal cancer) OR (anal cancer) OR (vulvar cancer)) AND ((pregnancy) OR (fertility preservation)) AND ((radiation) OR (radiotherapy)) are summarized in Table 1 [7–17]. While three cases of pregnancy after RT for vulvar cancer have been reported previously [7, 9, 13], to our knowledge, this appears to be the first reported case of recurrent vulvar cancer treated with RT following fertility preservation therapy.

**Table 1:** Pregnancy after pelvic radiotherapy in patients with vulvar, rectal, and anal cancers

	Year	Cancer	Age at RT	Type of RT	RT field and dose	Effective dose to the uterus	Age at pregnancy	Spontaneous or ART	Pregnancy outcome	Reference
1	2002	Vulvar	19	EBRT	Right hemipelvis 45 Gy & right inguinal 10.8 Gy	NA	25	Spontaneous	Preterm birth at 29 weeks	[7]
2	2007	Rectum	24	EBRT	Two lateral pelvis 50.4 Gy	NA	26	Spontaneous	Intrauterine fetal death at 21 weeks	[8]
3	2010	Vulvar	33	EBRT & brachytherapy	Perineum brachytherapy 20 Gy & EBRT 50 Gy	NA	35	Spontaneous	NA	[9]
4	2012	Anal	25	EBRT	Whole pelvis 50 Gy	30 Gy (cervix; 50 Gy)	26	Spontaneous	NA	[10]
5	2016	Rectum	33	EBRT	Primary tumor 50 Gy & pelvic lymph nodes 40 Gy	NA	36	Spontaneous	Twin, preterm, one baby died during delivery	[11]
6	2016	Anal	36	IMRT	Pelvis 59.4 Gy	NA	39	ART	Cesarean section at term	[12]
7	2016	Vulvar	17	EBRT	Vulvar and inguinal 45 Gy	NA	26	Spontaneous	Cesarean section at term	[13]
8	2017	Rectum	28	EBRT	Rectum 30.6 Gy	22.5 Gy	29	Spontaneous	Delivery at term	[14]
9	2020	Rectum	28	EBRT	NA	NA	30	Spontaneous	Cesarean section at 32 weeks	[15]
10	2021	Rectum	32	EBRT & brachytherapy	Whole pelvis EBRT 48.6 Gy & anal canal brachytherapy 10 Gy	16 Gy (cervix; 35 Gy)	42	ART (donation)	Twin, cesarean section at term	[16]
11	2024	Rectum	26	IMRT	Primary tumor 50 Gy & pelvic lymph nodes 45 Gy	Uterine fundus; 20-25 Gy, cervix; 45–47.5 Gy	33	ART	Cesarean section at term	[17]
12	Our case	Vulvar (recurrent)	36	IMRT	Vulvar, inguinal and pelvic lymph nodes 50.4 Gy	NA	36	ART	Artificial abortion at 6 weeks	−

RT; radiotherapy, EBRT; external beam radiotherapy, IMRT; intensity modulated radiotherapy, ART; assisted reproductive technology, NA; not available

Regarding uterine function after RT, pelvic irradiation can lead to fibrosis of the myometrium, reduced uterine volume and blood flow, and loss of the basal endometrium [3, 4]. However, Barnhill et al. reported that 7 out of 16 patients who received RT for cervical cancer exhibited endometrial proliferation following estrogen therapy [18]. De Hullu et al. observed hematometra in 2 of 4 young women who had received approximately 80 Gy for cervical cancer, suggesting partial preservation of endometrial function [19]. Furthermore, Rozen et al. reported a case of rectal cancer in which the endometrium became markedly thinned and unresponsive after RT, but thickened to a transferable level following high-dose estrogen therapy, leading to successful pregnancy and delivery [17].

These findings suggest that the endometrium is not always completely destroyed by RT. If partially functional endometrial tissue remains, regeneration may be stimulated through exogenous hormone therapy, even when spontaneous regrowth is limited. However, even if pregnancy is achieved after RT, the risk of obstetric complications due to impaired uterine function remains high, and careful perinatal management in a high-level medical facility is essential [20, 21].

In malignancies that do not directly involve the uterus, such as rectal, anal, or vulvar cancers, it is technically feasible to spare the uterus from radiation exposure (Table 1). In recent years, the introduction of IMRT has enabled precise control of the irradiation field and substantial reduction of scatter radiation to surrounding normal tissues [5]. Lee et al. reported a case in which successful pregnancy was achieved by minimizing radiation exposure to the uterine corpus during RT for cervical cancer [22]. Milgrom et al. noted that although complete avoidance of scatter radiation to the uterus is challenging, dose optimization and shaping can enhance the possibility of preserving uterine function [23]. Moreover, bladder filling during RT is commonly used to shift the uterus ventrally and out of the irradiation field [24], as was also performed in this case.

As a more challenging approach, uterine transposition prior to RT for non-gynecologic malignancies has been reported, and in one study, natural conception and live birth were achieved in 2 of 8 cases after repositioning the uterus post-treatment [25]. However, the feasibility of this technique for vulvar cancer remains unclear. There have been reports of successful natural pregnancies after ovarian transposition prior to RT [11, 15]. However, in the present case, ovarian transposition was not performed because it was anticipated that sufficient protection of the ovaries from scatter radiation would be difficult to achieve, and the surgical approach would have been more invasive due to a different operative field. Since scatter radiation to the ovaries cannot be fully predicted even with IMRT, embryo cryopreservation prior to treatment was considered the most practical and effective fertility preservation strategy.

The timing of embryo transfer should be carefully considered in light of the risk of disease recurrence. Although clear evidence regarding the second recurrence risk of vulvar cancer is lacking, previous studies have reported cumulative incidences of local recurrence after primary treatment of 5.9% at 2 years and 14.7% at 5 years [26]. Taking these data into account, together with the age-related decline in fertility, early embryo transfer after completion of treatment may be reasonable. In the present case, however, AML developed shortly after embryo transfer. Radiation-induced leukemia is recognized as a late complication of radiotherapy, but its incidence is very low. Large cohort studies of pelvic radiotherapy have shown a 72% increase in post-treatment leukemia; however, the peak incidence usually occurs 5–10 years after exposure, and the cumulative incidence remains 0.0022% [27]. In this case, AML occurred only a few months after completion of IMRT, which does not match the typical latency of radiation-induced leukemia. Therefore, while a causal relationship with radiotherapy cannot be excluded entirely, the temporal course suggests that the association is unclear and could not have been predicted.

In conclusion, this case provides a valuable clinical experience in which partial preservation of uterine function and successful pregnancy were achieved through technical modifications in IMRT and the use of hormone therapy. Given the nature of the disease, fertility preservation is often considered to be unfeasible. However, with thorough pre-treatment counseling and appropriate strategy, the possibility of preserving fertility can be maximized.

## References

[1] Abu-Rustum NR, Yashar CM, Arend R et al (2024) Vulvar cancer, version 3.2024, NCCN clinical practice guidelines in oncology. J Natl Compr Canc Netw 22:117–135. 10.6004/jnccn.2024.0013 38503056 10.6004/jnccn.2024.0013

[2] Lai J, Elleray R, Nordin A et al (2014) Vulval cancer incidence, mortality and survival in England: age-related trends. BJOG 121:728–738. 10.1111/1471-0528.12459 24148762 10.1111/1471-0528.12459

[3] Oktem O, Kim SS, Selek U et al (2018) Ovarian and uterine functions in female survivors of childhood cancers. Oncol 23:214–224. 10.1634/theoncologist.2017-0201 10.1634/theoncologist.2017-0201 PMC5813745 29158370

[4] Watanabe T, Matsubara S, Saito Y et al (2012) Pregnant woman with an extremely small uterus due to pelvic irradiation in childhood. J Obstet Gynaecol Res 38:559–561. 10.1111/j.1447-0756.2011.01730.x 22381106 10.1111/j.1447-0756.2011.01730.x

[5] Hong TS, Ritter MA, Tomé WA et al (2005) Intensity-modulated radiation therapy: emerging cancer treatment technology. Br J Cancer 92:1819–1824. 10.1038/sj.bjc.6602577 15856036 10.1038/sj.bjc.6602577 PMC2361760

[6] Dawson LA, Sharpe MB (2006) Image-guided radiotherapy: rationale, benefits, and limitations. Lancet Oncol 7:848–858. 10.1016/S1470-2045(06)70904-4 17012047 10.1016/S1470-2045(06)70904-4

[7] Serkies K, Wysocka B, Emerich J et al (2002) Salvage hemipelvis radiotherapy with fertility preservation in an adolescent with recurrent vulvar carcinoma. Gynecol Oncol 85:381–383. 10.1006/gyno.2002.6619 11972405 10.1006/gyno.2002.6619

[8] Kurt M, Uncu G, Cetintas SK et al (2007) Successful spontaneous pregnancy in a patient with rectal carcinoma treated with pelvic radiotherapy and concurrent chemotherapy: the unique role of laparoscopic lateral ovary transposition. Eur J Gynaecol Oncol 28:408–410 17966224

[9] Dicken CL, Lieman HJ, Dayal AK et al (2010) A multidisciplinary approach to fertility-sparing therapy for a rare vulvar tumor. Fertil Steril 93:67.e5–7. 10.1016/j.fertnstert.2009.07.1007 10.1016/j.fertnstert.2009.07.1007 19962143

[10] Hürmüz P, Sebag-Montefiore D, Byrne P et al (2012) Successful spontaneous pregnancy after pelvic chemoradiotherapy for anal cancer. Clin Oncol (R Coll Radiol) 24:455–457. 10.1016/j.clon.2012.03.006 22486987 10.1016/j.clon.2012.03.006

[11] Wald K, Easterling T, Swisher EM (2016) Spontaneous twin pregnancy after oophoropexy and pelvic radiation for rectal cancer. Obstet Gynecol 128:792–794. 10.1097/AOG.0000000000001516 27607863 10.1097/AOG.0000000000001516

[12] Köhler C, Marnitz S, Biel P et al (2016) Successful delivery in a 39-year-old patient with anal cancer after fertility-preserving surgery followed by primary chemoradiation and low anti-Mullerian hormone level. Oncology 91:295–298. 10.1159/000449416 27677176 10.1159/000449416

[13] Toriyabe K, Taniguchi H, Senda T et al (2016) Pregnancy and cesarean delivery after multimodal therapy for vulvar carcinoma: A case report. Mol Clin Oncol 5:583–586. 10.3892/mco.2016.1021 27900089 10.3892/mco.2016.1021 PMC5103870

[14] Hatayama Y, Aoki M, Kawaguchi H et al (2017) Safe and successful birth following pelvic radiotherapy for rectal mucosa-associated lymphoid tissue lymphoma: a case report. J Med Case Rep 11:26. 10.1186/s13256-016-1193-z 28143501 10.1186/s13256-016-1193-z PMC5286565

[15] Chae-Kim J, Hayslip CC Jr (2020) Fertility and endocrine preservation in the management of colorectal cancer in women. Dis Colon Rectum 63:723–726. 10.1097/DCR.0000000000001687 32384402 10.1097/DCR.0000000000001687

[16] Lohynska R, Jirkovska M, Novakova-Jiresova A et al (2021) Radiotherapy dose limit for uterus fertility sparing in curative chemoradiotherapy for rectal cancer. Biomed Pap Med Fac Univ Palacky Olomouc Czech Repub 165:99–101. 10.5507/bp.2020.039 32975243 10.5507/bp.2020.039

[17] Rozen G, Chander S, Polyakov A et al (2024) Live birth from ovarian grafted tissue after pelvic radiation for rectal cancer. F S Rep 5:214–218. 10.1016/j.xfre.2024.04.004 38983737 10.1016/j.xfre.2024.04.004 PMC11228876

[18] Barnhill D, Heller P, Dames J et al (1985) Persistence of endometrial activity after radiation therapy for cervical carcinoma. Obstet Gynecol 66:805–808 2999661

[19] de Hullu JA, Pras E, Hollema H et al (2005) Presentations of endometrial activity after curative radiotherapy for cervical cancer. Maturitas 51:172–176. 10.1016/j.maturitas.2004.07.005 15917158 10.1016/j.maturitas.2004.07.005

[20] Norwitz ER, Stern HM, Grier H et al (2001) Placenta percreta and uterine rupture associated with prior whole body radiation therapy. Obstet Gynecol 98:929–931. 10.1016/s0029-7844(01)01435-1 11704208 10.1016/s0029-7844(01)01435-1

[21] Baucom A, Herzog T, Jackson A et al (2021) A case of placenta previa with increta with a history of pelvic radiation. Gynecol Oncol Rep 37:100800. 10.1016/j.gore.2021.100800 34150975 10.1016/j.gore.2021.100800 PMC8192561

[22] Lee SW, Kim A, Lee SJ et al (2024) Intensity-modulated radiation therapy for uterine cervical cancer to reduce toxicity and enhance efficacy - an option or a must?: a narrative review. Cancer Res Treat 56:1–17. 10.4143/crt.2023.562 37654111 10.4143/crt.2023.562 PMC10789959

[23] Milgrom SA, Vargas HA, Sala E et al (2013) Acute effects of pelvic irradiation on the adult uterus revealed by dynamic contrast-enhanced MRI. Br J Radiol 86:20130334. 10.1259/bjr.20130334 24052311 10.1259/bjr.20130334 PMC3830434

[24] Eminowicz G, Motlib J, Khan S et al (2016) Pelvic organ motion during radiotherapy for cervical cancer: understanding patterns and recommended patient preparation. Clin Oncol (R Coll Radiol) 28:e85-91. 10.1016/j.clon.2016.04.044 27178706 10.1016/j.clon.2016.04.044

[25] Ribeiro R, Baiocchi G, Moretti-Marques R et al (2023) Uterine transposition for fertility and ovarian function preservation after radiotherapy. Int J Gynecol Cancer 33:1837–1842. 10.1136/ijgc-2023-004723 37898483 10.1136/ijgc-2023-004723

[26] Zach D, Åvall-Lundqvist E, Falconer H et al (2021) Patterns of recurrence and survival in vulvar cancer: a nationwide population-based study. Gynecol Oncol 161:748–754. 10.1016/j.ygyno.2021.03.013 33736857 10.1016/j.ygyno.2021.03.013

[27] Wright JD, St Clair CM, Deutsch I et al (2010) Pelvic radiotherapy and the risk of secondary leukemia and multiple myeloma. Cancer 116:2486–2492. 10.1002/cncr.25067 20209618 10.1002/cncr.25067

